# Continuous biosorption of acid red 27 azo dye by *Eichhornia crassipes* leaves in a packed-bed column

**DOI:** 10.1038/s41598-021-98034-4

**Published:** 2021-09-16

**Authors:** Allan Eduardo Ramírez-Rodríguez, Liliana Morales-Barrera, Eliseo Cristiani-Urbina

**Affiliations:** Departamento de Ingeniería Bioquímica, Escuela Nacional de Ciencias Biológicas, Instituto Politécnico Nacional, Avenida Wilfrido Massieu s/n, Unidad Profesional Adolfo López Mateos, Delegación Gustavo A. Madero, 07738 Mexico City, Mexico

**Keywords:** Environmental sciences, Materials science

## Abstract

In this work, the biosorption behavior of acid red 27 (AR27) dye using *Eichhornia crassipes* leaves (LECs) in a packed-bed column was investigated by varying relevant operational parameters and assessment of mathematical models. Results showed that the zero-charge point of LECs was 2.37 and that optima pH and volumetric flux of the influent solution for AR27 biosorption were 2.0 and $56.5\ \hbox {L}/\hbox {m}^{2}\cdot \hbox {h}$, respectively. The maximum specific and volumetric biosorption capacities were observed at influent AR27 concentrations and with LEC bed heights ranging between 50 and 400 mg/L and 2 and 8 cm, respectively. It was also found that if LEC bed height was increased and volumetric flux and AR27 concentration of the influent solution decreased, service and saturation time increased. Modeling results revealed that the Thomas, bed depth service time, Yoon–Nelson, dose-response, and logistic models accurately described the dynamic performance of the packed-bed column in terms of pH, AR27 concentration, and volumetric flux of influent AR27 solution, as well as that of LEC bed height. The findings revealed that LECs exhibited remarkable potential for the biosorption of AR27 from aqueous solutions in a packed-bed column and could potentially be useful for the treatment of AR27-laden wastewater.

## Introduction

Dyes are widely used in the textile, food, pharmaceutical, plastic, cosmetics, photographic and paper industries, with the textile industry representing the largest consumer of dyes^1,2^. Textile wastewaters may contain between 1 and 2500 mg/L of toxic synthetic non-biodegradable dyes^3^, toxic heavy metals, salts, cleansers, and corrosive-alkaline chemical agents^4^. Large amounts of synthetic dyes are disposed in water bodies due to the absence or inefficacy of wastewater treatment methods^3^. These generate environmental and public health problems because synthetic dyes affect the aesthetic properties of water, as well as aquatic flora and fauna, and are also toxic, mutagenic, teratogenic, carcinogenic, and harmful to human health^4,5^.

Acid red 27 (AR27) is a highly water-soluble anionic azo dye that is used extensively in industries to color natural and synthetic textiles, and produce leather, paper, food, beverages, confectionery, pharmaceuticals^6,7^, cosmetics, phenol-formaldehyde resins, and photographic images^8^. These industrial activities produce large amounts of wastewater contaminated with AR27. However, AR27 can be very hazardous to health, with effects that include respiratory problems, birth defects, allergies, tumors^7,9^, genotoxicity, cytotoxicity, embryotoxicity, mutagenicity, carcinogenicity^8,10^, and endocrine disruption^9^. This azo dye has been banned in the United States of America by the Food and Drug Administration (FDA) and its use is strictly controlled in many other countries^11,12^.

Furthermore, the discharge of AR27-containing effluents into water bodies reduces the penetration of sunlight, which affects the viability and photosynthetic process of aquatic plants, and deprives aquatic animals of the oxygen required for their vital functions^13^. Additionally, the complex chemical structure of AR27, which includes an azo group and conjugated benzene ring, renders it extremely recalcitrant to degradation, because of which it continues to persist in the environment^7,14^. Thus, it is crucial to remove AR27 from industrial effluents before their discharge into water bodies, to prevent it from having harmful effects on the environment and health.

Different approaches have been developed to achieve efficient removal of pollutants from industrial effluents, such as adsorption^15,16^, biosorption^17,18^, photocatalytic degradation^19,20^, reverse osmosis, nanofiltration^8,21^, ion exchange^8^, chemical oxidation^21,22^, chemical precipitation, coagulation, and biodegradation^21^. Conventional biological methods for the treatment of dye-contaminated wastewaters are not effective for the removal of dyes^21^ and can generate secondary products, which are far more toxic than the dye itself^23,24^. Major drawbacks to advanced chemical oxidation, chemical coagulation and precipitation, and photocatalytic oxidation methods are their high cost, complexity, and the generation of hazardous wastes^17,21^. Ion-exchange technique uses very high-cost resins that are quickly exhausted and difficult to regenerate^8^. Membrane-based separations consume an elevated amount of energy and require high-cost investments^21^; in addition, membranes become fouled very easily, and have a limited lifespan^8^. Adsorption/biosorption appears to be a more viable and attractive technology for the removal of dyes from industrial effluents because of its unique properties, which include effectiveness, ease of operation, flexibility, adaptability, simplicity of design, cost-effectiveness, efficiency, efficacy, insensitivity to toxic pollutants, eco-friendliness^9,21^, and adsorbent/biosorbent regeneration^18^.

AR27 adsorption/biosorption studies have only been conducted in batch systems using activated carbon prepared from coconut husk fiber, commercial activated carbon^19^, *Eichhornia crassipes*^13,25^, zeolitic imidazolate framework^22^, surfactant modified dull pink clay^8^, activated carbon web prepared from acrylic fibrous waste^26^, *Pisum sativum* peel, *Arachis hypogaea* shell^27^, and $\hbox {Fe}_{3}\hbox {O}_{4}/\hbox {MgO}$ nanoparticles^28^ as adsorbents/biosorbents. Batch systems are useful for the treatment of small volumes of effluents, and for determining the maximum dye adsorption/biosorption capacity of the adsorbent and effectiveness of the dye removal process in aqueous solutions^29,30^. However, the operational conditions for batch and continuous biosorption processes differ considerably; hence, the data collected in batch systems are commonly not applicable to industrial-scale continuous treatment systems^31^.

For the continuous treatment of industrial effluents on a large scale, it is preferable to use packed-bed column systems because of their high effectiveness, versatility, ease of use, low fixed and operating costs, and effluent quality^31,32^. Furthermore, the experimental data obtained using laboratory packed-bed columns are useful for the design, scale-up, optimization, and operation of continuous wastewater treatment processes on a pilot and industrial scale^30^. In addition, the modeling of the dynamic behavior of packed-bed columns is essential for identifying the relevant factors and mechanisms of the biosorption process, predicting future changes under different operating conditions, and the successful design, scale-up, and optimization of the biosorption processes^30,31^.

To the best of our knowledge, no study has reported on the adsorption/biosorption of the toxic AR27 dye in continuous systems; therefore, there are no reports on the kinetic modeling of the AR27 adsorption/biosorption process in packed-bed column systems. This study is the first to address these research gaps.

Recently, *Eichhornia crassipes* leaves (LECs) were reported to be among the most effective biosorbents available for the removal of AR27 in aqueous batch systems, because of their high capacity and rate of dye biosorption^25^. Characterization studies of AR27-unloaded LEC, AR27-loaded LEC, and AR27-desorbed LEC revealed that amide I and amide II functional groups, which are present in LEC proteins, are primarily responsible for the removal of AR27 from aqueous solutions by LECs^9,13^. Moreover, LECs can be reused in at least seven AR27 biosorption-desorption cycles in a batch system without resulting either in apparent physical change or damage to LECs or in a decrease in AR27 biosorption capacity and desorption efficiency or in loss of biosorbent^9^. Therefore, it is crucial to thoroughly investigate the ability of LECs to perform the continuous biosorption of AR27 in a packed-bed column system.

In the present work, the particles and packed beds of LECs were characterized, and their potential for AR27 biosorption from aqueous solutions was evaluated using a continuous packed-bed column system. The influence of fundamental design parameters, such as the pH, AR27 concentration, volumetric flux of the influent AR27 solution, and LEC bed height on the performance of the packed-bed column used for AR27 biosorption was examined. In addition, a mathematical modeling of the AR27 biosorption dynamics in the LEC packed-bed column was performed, and the experimental data were compared with values predicted using different breakthrough curve models.

## Methods

### Biosorbent

*Eichhornia crassipes* plants were collected from Xochimilco canals, in the San Gregorio Atlapulco area (19.2588422, -99.0848157), Mexico City, Mexico. The plant samples were identified by staff from the Herbarium (Dr. A. Novelo) of the National School of Biological Sciences, National Polytechnic Institute, Mexico City, Mexico (Collection ID: 2028).

The leaves were separated from the rest of the plant, washed with abundant quantities of tap water, followed by distilled water. The leaves were then cut into small pieces and oven-dried at $60\ ^{\circ }\hbox {C}$ for 36 h. The dried leaves were ground in a hammer mill and then sieved through standard ASTM sieves. A fraction of the particles with sizes ranging from 0.15 to 0.3 mm^25^ was recovered, and stored in screw-capped glass bottles for later use in biosorption experiments conducted in the packed-bed column reactor.

### AR27 stock and test solutions

A 2 g/L stock solution of AR27 (Sigma-Aldrich Chemicals, USA; $\hbox {purity} = 95\%$) was prepared and diluted with distilled water to prepare the AR27 test solutions. The pH of each AR27 test solution was adjusted with 0.1 M HCl or 0.01 M NaOH to obtain solutions with the desired pH value.

### Biosorbent characterization

The proximate chemical composition of LECs was determined by applying the methodology outlined in the Official Methods of Analysis of AOAC International^33^. The chemical composition of LECs was previously reported to be as follows: total protein, 33.34% $\pm \,{0.05\%}$; total ash, 14.93% $\pm \,{0.01\%}$; ether extract, 4.89% $\pm \,{0.25\%}$; crude fiber, 12.54% $\pm \,{0.35\%}$; and nitrogen-free extract, 34.29% $\pm \,{0.72\%}$^13^. Thus, LECs are rich in carbohydrates and proteins, and have low levels of lipid and inorganic matter.

To analyze their zeta potential, 1 mg/L LEC suspensions with different pHs (1.5, 2, 2.5, 3, 4, 6, 8, 10, and 12) were prepared using distilled water, and examined using a Zetasizer Nano-25 analyzer (Malvern Instruments Ltd., Malvern, England) at room temperature ($20\, \pm \,{2}^{\circ }\hbox {C}$). The LEC particle size was determined using the same equipment using 1 mg/L of the LEC suspension at a pH of 6.0.

The LEC pore diameter and specific surface area were determined from nitrogen adsorption-desorption isotherms at 77 K using a Quantachrome ASiQwin gas sorption analyzer (Quantachrome Instruments, Boynton Beach, FL.). The LEC particles were degassed with nitrogen at 373 K. The LEC specific surface area was evaluated using the Brunauer, Emmett and Teller (BET) multipoint method^34^, and the pore size distribution was obtained by applying the Barrett, Joyner and Halenda (BJH) method^35^.

The porosity and bulk density values of the LEC packed bed were determined in triplicate, via the procedure described by Atarés^36^.

### Continuous biosorption studies of AR27 in a packed-bed column

**Figure 1:**
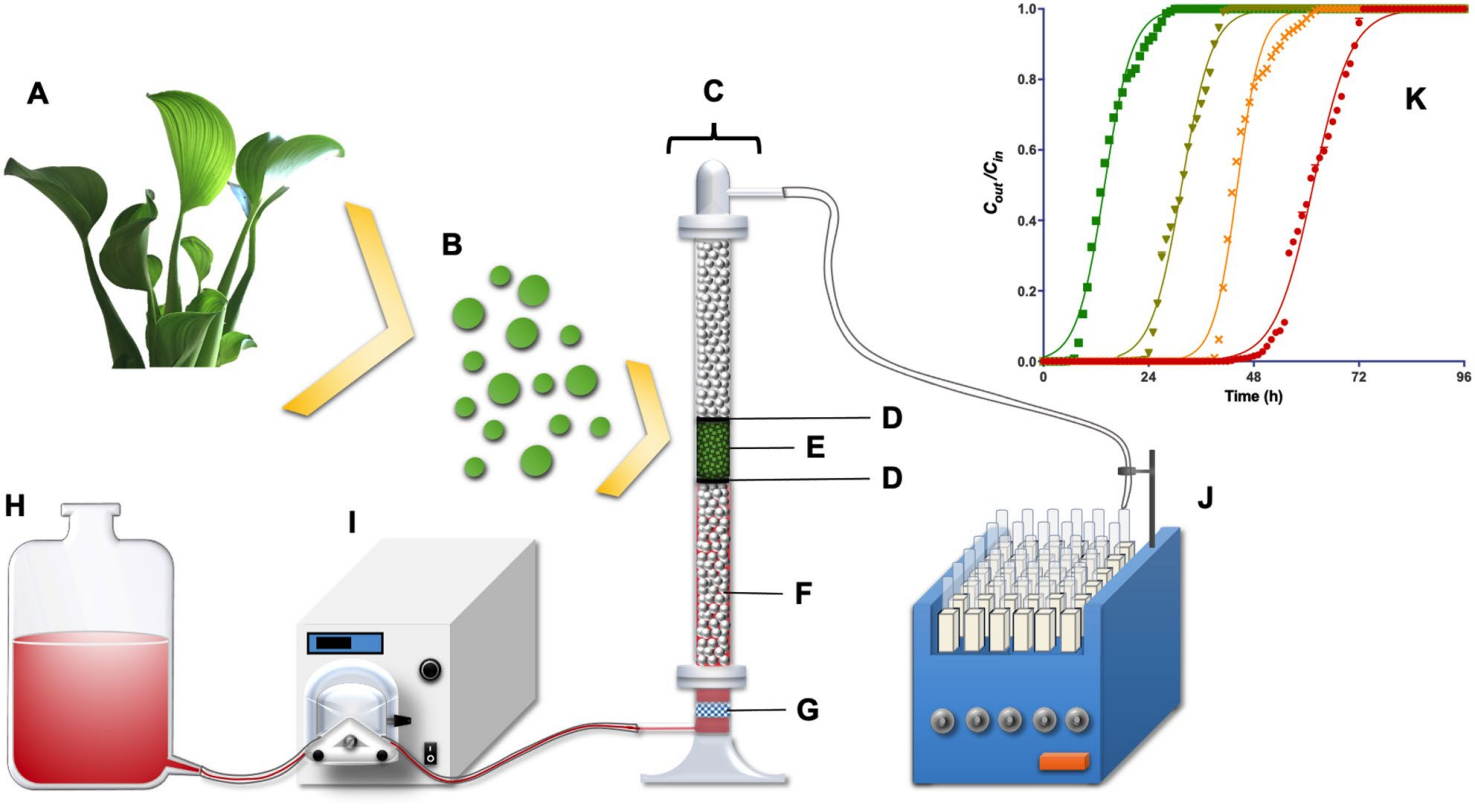
Graphical abstract: schematic representation of the experimental packed-bed column system (**A**) LECs; (**B**) LEC particles; (**C**) cylindrical column; (**D**) 200-mesh stainless steel sieve; (**E**) LEC packed bed; (**F**) glass beads; (**G**) liquid diffuser; (**H**) AR27 solution reservoir; (**I**) peristaltic pump; (**J**) fraction collector; (**K**) breakthrough curves.

Continuous AR27 biosorption experiments were performed in a 20-cm-high cylindrical glass column with an internal diameter of 1.3 cm (Fig. 1). Certain amounts of LECs were placed inside the column to obtain biosorbent beds with different heights. Spherical glass beads with a diameter of 0.3 cm were placed on the top and bottom of LEC packed beds with heights of 4 and 7 cm, respectively. The glass beads at the bottom of the LEC packed bed served as a support for the LEC bed and enabled the inlet AR27 solution to be evenly distributed throughout the entire cross section of the column. The glass beads on the top of the LEC bed prevented the loss of LEC biosorbent particles. Additionally, a 200-mesh stainless steel sieve was placed between the glass beads and LEC biomass to avoid the loss of biosorbent.

A liquid diffuser made of sintered glass was placed at the bottom of the column to aid the even distribution of the inlet AR27 solution throughout the cross-section of the column. The liquid diffuser was connected to a peristaltic pump (Masterflex L/S) using silicone tubing. AR27 solutions with known concentrations and pHs were pumped up through the biosorbent bed at the desired flow rate, at room temperature ($20 \pm {1}^{\circ }\hbox {C}$).

To explore the effect of the pH of the solution on AR27 biosorption, experiments were conducted using inlet dye solutions with varied pHs in the range of 1.5 to 6 (1.5, 2, 3, 4, 5, and 6), and an influent solution with a constant AR27 concentration of 50 mg/L, while ensuring that the volumetric flux of the AR27 solution was $56.5\ \hbox {L}/\hbox {m}^{2}\cdot \hbox {h}$ (corresponding to a flow rate of 7.5 mL/h) and LEC bed height was 2 cm (corresponding to a LEC mass of 0.5 g). The optimal pH for AR27 biosorption was selected based on the results, and solutions with the optimal pH were prepared and used in subsequent studies.

Likewise, four different volumetric fluxes, 37.67, 56.5, 75.34, and $113\ \hbox {L}/\hbox {m}^{2}\cdot \hbox {h}$ (corresponding to flow rates of 5, 7.5, 10, and 15 mL/h), of AR27 solution were analyzed, to determine their effect on the biosorption of AR27 by the LEC packed bed, in which an inlet AR27 concentration of 50 mg/L and LEC bed height of 2 cm (0.5 g) were used.

To investigate the effect of the inlet AR27 concentration on dye biosorption, experiments were performed using an influent solution with a volumetric flux of $56.5\ \hbox {L}/\hbox {m}^{2}\cdot \hbox {h}$, a bed height of 2 cm (0.5 g), and dye concentrations of 30, 50, 75, 100, 200, and 400 mg/L.

When the effect of the LEC bed height was examined, the volumetric flux of the AR27 solution was $56.5\ \hbox {L}/\hbox {m}^{2}\cdot \hbox {h}$, influent AR27 concentration was 200 mg/L, and the LEC bed heights were 2, 4, 6, and 8 cm, which corresponded to LEC masses of 0.5, 1, 1.5, and 2 g, respectively.

A fraction collector (LKB Bromma, Ltd.) was used to collect effluent samples from the packed-bed column reactor, which were centrifuged at 3000 rpm for 10 min. Then, supernatants were analyzed using a Thermo Fisher Scientific Evolution 201 spectrophotometer at a wavelength of 520 nm to determine the AR27 concentration^13^.

Controls were run without LEC biomass under the same operating conditions as those used in the continuous AR27 biosorption experiments, to detect possible dye losses due to AR27 adsorption on the glass, photolysis, and/or precipitation. The AR27 concentrations of the influent and effluent solutions did not show statistically significant differences in any of the LEC-free controls assayed, which indicates that the decrease in the AR27 concentration observed in the effluents from the continuous biosorption experiments with LEC biomass was solely attributable to the biosorbent.

### Analysis and mathematical modeling of AR27 continuous biosorption data

The continuous biosorption of AR27 on the LEC packed-bed column was studied in terms of the breakthrough curves, which were expressed by the relationship between the outlet AR27 concentration ($\hbox {C}_{out}$, mg/L) and inlet AR27 concentration ($\hbox {C}_{in}$, mg/L) as a function of time ($\hbox {C}_{out}/\hbox {C}_{in}$ vs. time, *t*). The specific biosorption capacity (which is usually simply referred to as the capacity of biosorption) of AR27 (q, mg/g) was calculated using Eq. 1^32^:

1 $$\begin{aligned} q = \frac{\hbox {Q}\,\hbox {C}_{in}}{1000\,\hbox {m}} \int _{t=0}^{t=ts} \left( 1-\frac{\hbox {C}_{out}}{\hbox {C}_{in}}\right) \;dt \end{aligned}$$

here Q is the flow rate of the inlet AR27 solution (mL/h), m is the LEC mass packed in the column (g), and $\hbox {t}_{\mathrm{s}}$ is the operating time of the packed-bed column, in which the ratio $\hbox {C}_{out}/\hbox {C}_{in}$ was equal to 1.0 (h).

The volumetric biosorption capacity ($\hbox {N}_{\mathrm{v}}$, mg/L) was estimated based on Eq. 2 as follows^32^:

2 $$\begin{aligned} \hbox {N}_{\mathrm{v}} = q\, \rho _{\mathrm{b}} \end{aligned}$$

here $\rho _b$ is the packed bed bulk density (g/L).

The successful optimization of the design and operating conditions of a packed-bed column biosorption process requires the ability to predict the biosorption breakthrough curve^37,38^.

The Thomas, Yoon–Nelson, Bed Depth Service Time (BDST), modified Dose-Response, and logistic models were used in the present study to match the experimental data, and predict the dynamic behavior during the process of AR27 biosorption on LEC in the packed-bed column at different pHs, volumetric fluxes, AR27 concentrations of the influent solution, and LEC bed heights.

The Thomas non-linear model has the following simplified form (Eq. 3)^30^:

3 $$\begin{aligned} \frac{\hbox {C}_{out}}{\hbox {C}_{in}} =\frac{1}{1+e^{\frac{K_{Th} q \,m}{Q}-k_{Th}C_{in}t}} \end{aligned}$$

here $\hbox {C}_{in}$ is the AR27 concentration in the influent solution (mg/L), $\hbox {C}_{out}$ is the AR27 concentration in the effluent solution (mg/L) at time t (h), q is the maximum biosorption capacity (mg/g), $\hbox {k}_{\mathrm{Th}}$ is the Thomas model rate constant (L/h$\cdot$mg), m is the mass of the biosorbent in the packed-bed column (g), and Q is the flow rate of the inlet AR27 solution (L/h).

The bed depth service time (BDST) model can be expressed in terms of Eq. 4^39^:

4 $$\begin{aligned} \frac{\hbox {C}_{out}}{\hbox {C}_{in}} =\frac{1}{1+e^{\left( N_{vBDST}\,k_{BDST} \frac{Z}{v}\right) }-(k_{BDST}C_{in}t)} \end{aligned}$$

here $\hbox {N}_{\mathrm{vBDST}}$ is the volumetric biosorption capacity predicted by the BDST model (mg/L), $\hbox {k}_{\mathrm{BDST}}$ is the biosorption rate constant (L/mg$\cdot$h), v is the superficial velocity (cm/h), and Z is the column bed depth (cm).

The non-linear form of the Yoon–Nelson model is expressed as follows (Eq. 5)^40^:

5 $$\begin{aligned} \frac{\hbox {C}_{out}}{\hbox {C}_{in}} =\frac{1}{1+e\,^{k_{YN}(\tau _{YN}-t)}} \end{aligned}$$

here $\hbox {k}_{\mathrm{YN}}$ is the rate constant (1/h), and $\tau _{\mathrm{YN}}$ is the operating time required to reach 50% AR27 breakthrough ($\hbox {C}_{out}/\hbox {C}_{in} = 0.5$) (h).

The modified dose-response model can be expressed in terms of Eq. 6^41^:

6 $$\begin{aligned} \frac{\hbox {C}_{out}}{\hbox {C}_{in}} =1-\frac{1}{1+\left( \frac{t}{\tau _{DR}}\right) ^\alpha } \end{aligned}$$

here $\alpha$ and $\tau _{\mathrm{DR}}$ are constants of the modified dose-response model. $\tau _{\mathrm{DR}}$ is the operating time at which the effluent AR27 concentration is 50% of the influent dye concentration ($\hbox {C}_{out}/\hbox {C}_{in} = 0.5$) (h).

It has been claimed that the Bohart–Adams (variant of the BDST model), Thomas, and Yoon–Nelson models can be expressed in terms of the logistic model, which is expressed as follows (Eq. 7)^42^:

7 $$\begin{aligned} \frac{\hbox {C}_{out}}{\hbox {C}_{in}}=\frac{1}{1+e\,^{(a-b\,t)}} \end{aligned}$$

here a and b are logistic model constants, which are related to the Thomas, BDST, and Yoon–Nelson model constants, as shown in Table 1^42^.

**Table 1:** Parameters of the Thomas, BDST, and Yoon–Nelson models, expressed in terms of the logistic model’s parameters a and b.

Model	a	b
Thomas	$\frac{\hbox {k}_{\mathrm{Th}}\,\hbox {q}\,\hbox {m}}{\hbox {Q}}$	$\hbox {k}_{\mathrm{Th}}\,\hbox {C}_{0}$
BDST	$\frac{\hbox {k}_{\mathrm{BDST}}\,\hbox {N}_{\mathrm{VBDST}}\,\hbox {Z}}{\hbox {v}}$	$\hbox {k}_{\mathrm{BDST}}\,\hbox {C}_{0}$
Yoon–Nelson	$\hbox {k}_{\mathrm{YN}}\,\tau _{\mathrm{YN}}$	$\hbox {k}_{\mathrm{YN}}$

### Determination of the breakthrough curve parameters and statistical analysis

To ensure result reproducibility and perform statistical analysis, continuous AR27 biosorption experiments were carried out in triplicate. The mean ± standard deviation values are reported in this work.

GraphPad Prism version 8.0 software (GraphPad Software, Inc., La Jolla California, USA) was employed both to perform the statistical analysis of the AR27 biosorption data and determine the kinetic parameters of the packed-bed column models. The AR27 biosorption data were subjected to analysis of variance (ANOVA) and Tukey’s test with a 95% confidence level.

The kinetic parameters of packed-bed column models were determined using non-linear regression analysis. The models that were best adjusted to the experimental data were selected, to ensure that the coefficient of determination ($\hbox {R}^{2}$) would be maximal, and Akaike information criterion (AIC) and root mean squared error (RMSE) values were minimal, while the 95% confidence intervals remained the narrowest.

All methods were performed in accordance with the relevant guidelines and regulations.

## Results and discussion

### Characterization of particles and packed LEC beds

The average specific surface area, particle size, and pore size values of LECs were found to be 3.359 $\pm {0.168}$ $\hbox {m}^2/\hbox {g}$, 0.246 $\pm {0.012}$ mm, and 2.249 $\pm {0.113}$ nm, respectively; these properties are considered to be important and indicative of biosorbent effectiveness. The average pore size of LECs (2.249 nm) indicates that it is a mesoporous biomaterial.

**Figure 2:**
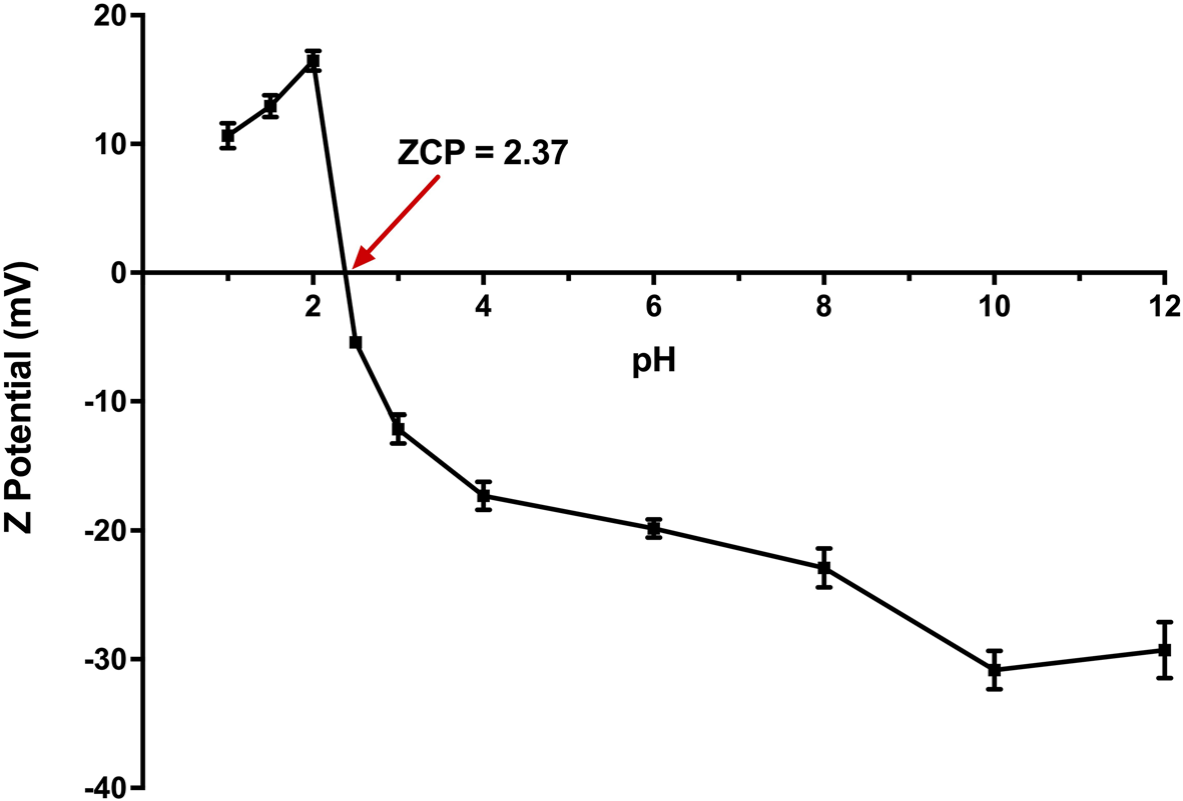
Variation in the LEC zeta potential with a change in the solution pH.

The variation in the LEC zeta potential with respect to the solution pH is shown in Fig. 2. The solution pH at which the zeta potential of the biosorbent surface is zero is known as the zero-charge point (ZCP) or isoelectric point; at this pH, the net electrical charge on the biosorbent surface is zero^43^. The zero-charge point of the LEC was found to be 2.37 (Fig. 2).

The zeta potential of the LEC surface was positive at solution pH values below 2.37, and negative at higher values, indicating that the LEC surface is positively charged in solutions with a pH below 2.37, and is negatively charged in solutions with a pH greater than 2.37. The highest LEC zeta potential was 16.47 mV and was observed at a solution pH of 2.0; therefore, at this solution pH, the LEC surface is more positively charged.

The porosity and bulk density of the LEC packed bed was 25% and 0.188 $\pm {0.009}\ \hbox {g}/\hbox {cm}^3$, respectively.

### Influence of the inlet solution pH on AR27 biosorption

The influence of solution pH on the biosorption of an adsorbate has been extensively studied in batch systems; however, very few studies have examined the effect of pH on the biosorption of the adsorbate of interest in continuous systems^32,44^.

**Figure 3:**
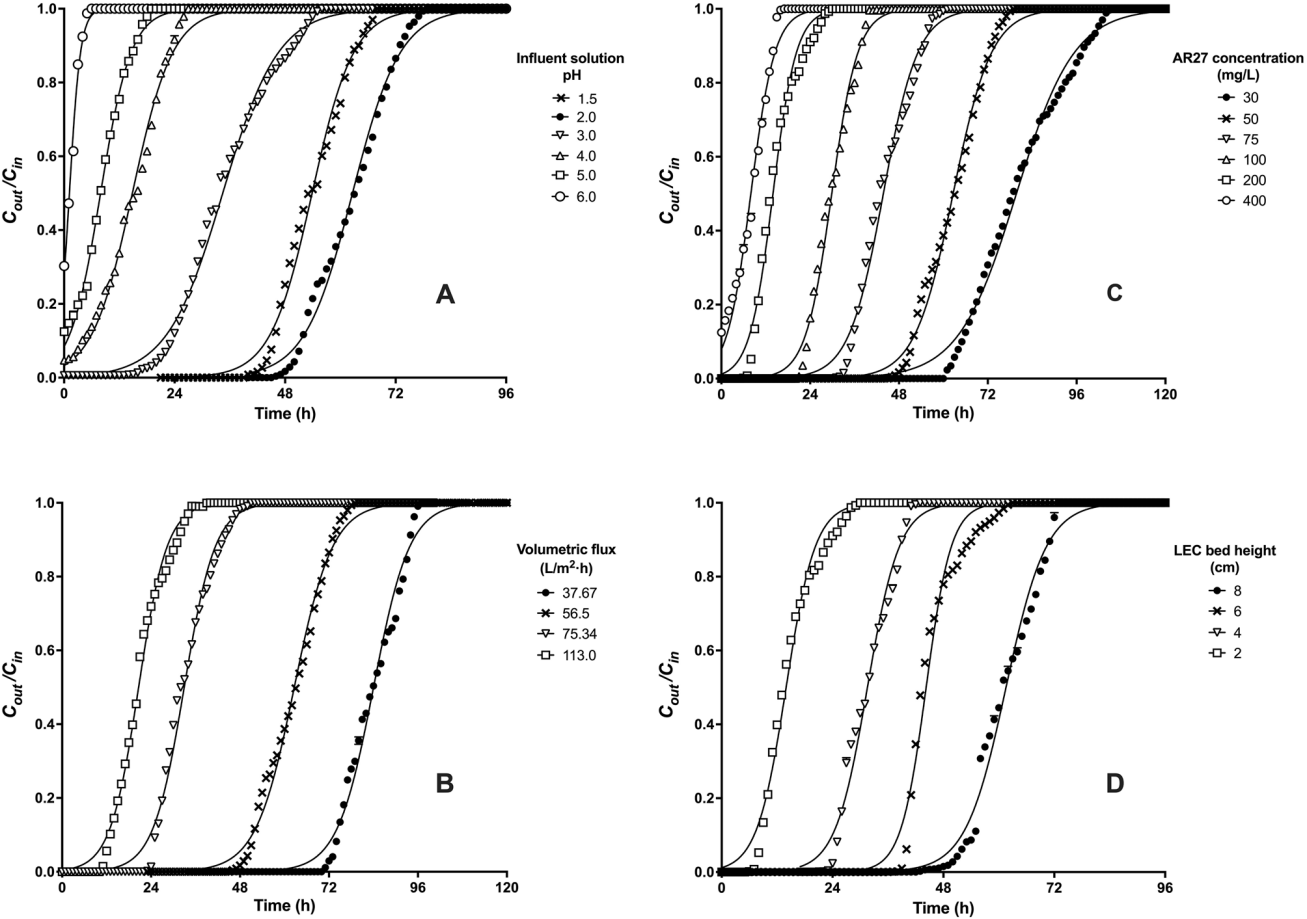
Effect of (**A**) influent solution pH, (**B**) influent volumetric flux, (**C**) influent AR27 concentration, and (D) LEC bed height on the dye biosorption breakthrough curve.

Figure 3A displays the experimental breakthrough curves for AR27 biosorption with influent AR27 solutions with pHs in the range of 1.5–6.0. It was found that the pH of the inlet solution significantly affected the gradient and shape of breakthrough curves, and therefore the AR27 concentration of effluents and AR27 biosorption capacity of the packed-bed column.

The lowest effluent AR27 concentrations were obtained during the first hours of operation of the packed-bed column; afterwards, the AR27 concentration of effluents gradually increased with an increase in the operating time, suggesting a decrease in the AR27 biosorption capacity due to the progressive depletion of the active binding biosorption sites on the surface of the LEC biomass.

No measurable AR27 was detected in the column effluents during the first 45, 39, and 10 h of operation of the packed-bed column when the influent solution pHs were 2, 1.5, and 3, respectively. In contrast, AR27 dye was detected from the start of operation of the packed-bed column at influent solution pHs of 4, 5, and 6.

As shown in Fig. 3A, the service and saturation time values of the packed-bed column were longer at pH 2.0, followed by those observed at pHs 1.5, 3.0, 4.0, 5.0, and 6.0.

Similarly, from the beginning to the end of the column operation period, the lowest effluent AR27 concentrations were observed at inlet AR27 solution pHs of 2.0, and 1.5. Likewise, the maximal mass of AR27 removed ($\hbox {m}_{\mathrm{r}}$), volumetric biosorption capacity ($\hbox {N}_{\mathrm{v}}$), and specific biosorption capacity (q) of AR27 were observed with an influent dye solution with a pH of 2, followed by those observed at pHs of 1.5, 3, 4, 5, and 6 (Table 2), with significant differences at all tested pHs ($p < 0.05$). These findings concur well with those of the batch biosorption assays reported in a previous article^25^.

**Table 2:** Kinetic parameter values from various breakthrough curve models for AR27 biosorption onto LEC using influent solutions with different pHs.

	pH					
	1.5	2	3	4	5	6
**Experimental**						
$\hbox {m}_{\mathrm{r}}$ (mg)	$30.25 \pm 1.51$	$33.63 \pm 1.68$	$22.25 \pm 1.13$	5$.63 \pm 0.28$	$2.93 \pm 0.15$	$0.42 \pm 0.02$
q (mg/g)	$60.5 \pm 3.02$	$67.25 \pm 3.37$	$45.125 \pm 2.26$	$11.25 \pm 0.56$	$5.85 \pm 0.29$	$0.84 \pm 0.04$
$\hbox {N}_{\mathrm{v}}$ (mg/L)	$11{,}381 \pm 569.0$	$12{,}611 \pm 630.5$	$8{,}635 \pm 431.75$	$2{,}115 \pm 105.75$	$1{,}100 \pm 55$	$157.92 \pm 7.90$
$\tau$ (h)	$54 \pm 2.7$	$63 \pm 3.15$	$33.5 \pm 1.68$	$15 \pm 0.75$	$7.8 \pm 0.39$	$1.12 \pm 0.06$
**Thomas**						
q (mg/g)	$60.39 \pm 0.11$	$66.92 \pm 0.13$	$45.84 \pm 0.1$	$11.12 \pm 0.12$	$5.94 \pm 0.04$	$0.86 \pm 0.02$
$\hbox {k}_{\mathrm{Th}}$ (L/mg h)	$0.004 \pm 0.001$	$0.004 \pm 0.001$	$0.003 \pm 0.001$	$0.004 \pm 0.001$	$0.006 \pm 0.001$	$0.016 \pm 0.001$
$\hbox {R}^{2}$	0.997	0.995	0.997	0.995	0.998	0.994
RMSE	0.207	0.185	0.177	0.132	0.044	0.023
AIC	− 2714	− 2476	− 2771	− 874.3	− 3276	− 3514
**Bed depth service time (BDST)**						
$\hbox {N}_{\mathrm{vBDST}}$ (mg/L)	$11{,}368 \pm 20.02$	$12{,}597 \pm 23.84$	$8{,}627 \pm 18.80$	$2{,}095 \pm 23.54$	$1{,}116 \pm 7.02$	$161.68 \pm 3.30$
$\hbox {k}_{\mathrm{BDST}}$ (L/ mg h)	$0.004 \pm 0.001$	$0.004 \pm 0.001$	$0.003 \pm 0.001$	$0.004 \pm 0.001$	$0.006 \pm 0.001$	$0.016 \pm 0.001$
$\hbox {R}^{2}$	0.997	0.995	0.997	0.9952	0.998	0.994
RMSE	0.207	0.185	0.177	0.132	0.044	0.023
AIC	− 2714	− 2610	− 2771	− 874.3	− 3276	− 3514
**Yoon–Nelson**						
$\tau _{\mathrm{YN}}$ (h)	$53.86 \pm 0.14$	$62.55 \pm 0.17$	$34.45 \pm 0.13$	$14.83 \pm 0.17$	$7.923 \pm 0.05$	$1.151 \pm 0.02$
$\hbox {k}_{\mathrm{YN}}$ (1/h)	$0.223 \pm 0.006$	$0.196 \pm 0.006$	$0.166 \pm 0.003$	$0.227 \pm 0.008$	$0.301 \pm 0.004$	$0.826 \pm 0.017$
$\hbox {R}^{2}$	0.997	0.995	0.997	0.992	0.998	0.994
RMSE	0.207	0.185	0.177	0.132	0.044	0.023
AIC	− 2714	− 24,610	− 2771	− 874.3	− 3276	− 3514
**Modified dose-response**						
$\tau _{\mathrm{DR}}$ (h)	$53.56 \pm 0.14$	$62.31 \pm 0.19$	$33.71 \pm 0.11$	$14.17 \pm 0.17$	$7.45 \pm 0.15$	$1.2 \pm 0.07$
$\alpha$	$11.94 \pm 0.32$	$12.22 \pm 0.39$	$5.71 \pm 0.10$	$3.60 \pm 0.26$	$4.83 \pm 0.13$	$2.98 \pm 0.17$
$\mathrm{R}^{2}$	0.997	0.994	0.998	0.965	0.976	0.872
RMSE	0.195	0.237	0.131	0.560	0.461	0.463
AIC	− 2736	− 2548	− 2879	− 689.2	− 2422	− 2420
**Logistic equation**						
a	$11.94 \pm 0.32$	$12.22 \pm 0.39$	$5.71 \pm 0.10$	$3.60 \pm 0.27$	$4.83 \pm 0.13$	$2.98 \pm 0.17$
b	$0.222 \pm 0.006$	$0.193 \pm 0.006$	$0.166 \pm 0.003$	$0.227 \pm 0.008$	$0.301 \pm 0.004$	$0.826 \pm 0.17$
$\hbox {R}^{2}$	0.996	0.994	0.997	0.992	0.998	0.994
RMSE	0.207	0.185	0.177	0.132	0.044	0.023
AIC	− 2013	− 1423	− 2771	− 874.3	− 3276	− 3514

The dependence of AR27 biosorption on the influent solution pH can be explained by the LEC surface zeta potential value (Fig. 2). The zeta potential of the biosorbent surface is positive when the solution pHs are 1.5, and 2.0, which indicates that at these solution pHs, the LEC surface displays a net positive charge. Therefore, at these pHs, the LEC surface has a higher AR27 biosorption capacity because of the large electrostatic attractive forces between the negative charges on the anionic AR27 dye and positive charges on the biosorbent surface. Furthermore, the net positive charge of the LEC surface was higher at a pH of 2 (+16.47 mV) than that observed at a pH of 1.5 (12.93 mV). Thus, the LEC surface had a higher AR27 biosorption capacity at pH 2.0 than at pH 1.5, probably due to the presence of higher electrostatic attractive forces on its surface.

In contrast, the surface zeta potential of the LECs was negative when the pH of the AR27 solution was greater than the zero-charge point pH (i.e., greater than pH 2.37). Likewise, the zeta potential became more negative as the pH of the solution increased from the zero-charge point pH to pH 10, which indicates that the net charge on the LEC surface became more negative as the solution pH increased. Therefore, the decrease observed in the AR27 biosorption capacity in solutions with pHs ranging from 3 to 6 was attributable to the fact that when the solution pH increased, the electrostatic repulsive forces between the negative charges on the anionic AR27 dye and negative charges on the LEC surface increased

It is apparent from our results that the optimal pH for continuous AR27 biosorption was 2.0, since at this pH, the specific (67.25 mg/g) and volumetric (12,611 mg/L) AR27 biosorption capacities were maximal, and the operating time of the packed-bed column was longer. Therefore, subsequent studies were carried out using an influent AR27 solution with a pH of 2.0.

### Influence of the influent volumetric flux on AR27 biosorption

Figure 3B shows experimental AR27 breakthrough curves, derived using influent dye solutions with different volumetric fluxes, namely 37.67, 56.5, 75.34, and 113 $\hbox {L}/\hbox {m}^{2}\, \hbox {h}$ (corresponding to flow rates of 5, 7.5, 10, and 15 mL/h), while the LEC packed-bed height (2 cm), inlet solution pH (2.0), and influent AR27 concentration (50 mg/L) values remained constant. AR27 dye was not detected in the outflow effluents during the first 70, 45, 22, and 11 h of packed-bed column operation, when the influent volumetric fluxes were 37.67, 56.5, 75.34, and 113 $\hbox {L}/\hbox {m}^{2}\, \hbox {h}$, respectively, indicating that the column service time decreased as the volumetric flux of the influent solution increased. Subsequently, the AR27 dye concentration in the outflow effluent increased as the volumetric flux of the influent solution increased, which indicated a decrease in the AR27 biosorption capacity of the LEC bed, attributable to an increased depletion of the active sites for AR27 dye biosorption. In addition, it is evident that the saturation time decreased as the influent volumetric flux increased.

The above results clearly show that the service and saturation time were longer for the lowest volumetric flux tested and shorter for the highest volumetric flux of the influent AR27 solution assayed. Similar results were found previously for the biosorption of yellow dye tartrazine onto *Moringa oleifera* seeds^45^, remazol reactive dyes onto *Rhizopus arrhizus* NCIM 997^46^, acid blue 25 onto NaOH-treated fallen *Ficus racemosa* leaves^47^, and reactive blue BF-5G onto malt bagasse^48^ in packed-bed columns.

Similarly, the highest mass of removed AR27, and highest specific and volumetric biosorption capacities of AR27 were observed at volumetric fluxes of 37.67 $\hbox {L}/\hbox {m}^{2}\, \hbox {h}$ (31 $\pm {1.55}\ \hbox {g}$, 62 $\pm {3.1}\ \hbox {mg}/\hbox {g}$, and 11,668 $\pm {583.4}\ \hbox {mg}/\hbox {L}$, respectively) and 56.5 $\hbox {L}/\hbox {m}^{2}\, \hbox {h}$ (33.63 $\pm {1.68}\ \hbox {g}$, 67.25 $\pm {3.36}\ \hbox {mg}/\hbox {g}$, and 12,611 $\pm {630.55}\ \hbox {mg}/\hbox {L}$, respectively) (Table 3), with no statistically significant difference in either the masses of AR27 removed or volumetric and specific capacities observed with the two different volumetric fluxes ($\hbox {p} > 0.05$). These results indicate that the maximum biosorption capacity of the packed-bed column is not used efficiently at very high volumetric fluxes of influent AR27 solution.

**Table 3:** Kinetic parameter values from various breakthrough curve models for AR27 biosorption onto LEC using influent solutions with different volumetric fluxes.

	Volumetric flux ($\hbox {L}/\hbox {m}^{2}\,\hbox {h}$)			
	37.67	56.5	75.34	113
**Experimental**				
$\hbox {m}_{\mathrm{r}}$ (mg)	31 ± 1.55	33.63 ± 1.68	26 ± 1.3	25.38 ± 1.27
q (mg/g)	62 ± 3.1	67.25 ± 3.36	52 ± 2.6	50.75 ± 2.54
$\hbox {N}_{\mathrm{v}}$ (mg/L)	11,668 ± 583.4	12,611 ± 630.55	9,911 ± 495.55	9,591 ± 479.55
$\tau$ (h)	84 ± 4.2	63 ± 3.15	32 ± 1.6	20.5 ± 1.025
**Thomas**				
q (mg/g)	62.02 ± 0.11	66.92 ± 0.14	52.95 ± 0.12	50.96 ± 0.14
$\hbox {k}_{\mathrm{Th}}$ (L/mg h)	0.004 ± 0.001	0.004 ± 0.001	0.005 ± 0.001	0.005 ± 0.001
$\hbox {R}^{2}$	0.987	0.996	0.997	0.976
RMSE	0.291	0.272	0.249	0.147
AIC	− 1033	− 2940	− 2981	− 3193
**Bed depth service time (BDST)**				
$\hbox {N}_{\mathrm{vBDST}}$ (mg/L)	11,675 ± 15.35	12,597 ± 19.37	9,966 ± 22.54	9,591 ± 25.94
$\hbox {k}_{\mathrm{BDST}}$ (L/mg h)	0.004 ± 0.001	0.004 ± 0.001	0.005 ± 0.001	0.005 ± 0.001
$\hbox {R}^{2}$	0.992	0.995	0.997	0.997
RMSE	0.310	0.187	0.229	0.147
AIC	− 2917	− 2791	− 2981	− 3193
**Yoon–Nelson**				
$\tau _{\mathrm{YN}}$ (h)	84.05 ± 0.23	62.55 ± 0.14	32.95 ± 0.12	20.64 ± 0.09
$\hbox {k}_{\mathrm{YN}}$ (1/h)	0.203 ± 0.009	0.196 ± 0.005	0.238 ± 0.006	0.260 ± 0.006
$\hbox {R}^{2}$	0.987	0.996	0.997	0.997
RMSE	0.291	0.272	0.249	0.147
AIC	− 1033	− 2940	− 2981	− 3193
**Modified dose-response**				
$\tau _{\mathrm{DR}}$ (h)	83.8 ± 0.16	62.31 ± 0.15	32.54 ± 0.09	20.15 ± 0.07
$\alpha$	17.24 ± 0.51	12.1 ± 0.32	7.82 ± 0.15	5.37 ± 0.08
$\hbox {R}^{2}$	0.995	0.996	0.998	0.999
RMSE	0.319	0.240	0.14	0.077
AIC	− 2396	− 2231	− 3213	− 3455
**Logistic equation**				
a	17.23 ± 0.51	12.08 ± 0.283	7.86 ± 0.2	5.37 ± 0.12
b	0.205 ± 0.006	0.193 ± 0.005	0.239 ± 0.006	0.2603 ± 0.006
$\hbox {R}^{2}$	0.995	0.997	0.997	0.997
RMSE	0.310	0.187	0.229	0.147
AIC	− 2406	− 2308	− 3014	− 3193

The decrease in the service and saturation time, AR27 mass removed, and specific and volumetric biosorption capacity with an increase in the influent volumetric flux may be attributable to the fact that the contact time between the AR27 azo dye and the LEC biomass in the column decreases as the volumetric flux increases. Thus, the AR27 molecules do not have enough time to attach to the biosorption sites present on the LEC surface or diffuse into the LEC pores, and leave the packed-bed column at higher concentrations, before biosorption equilibrium can be attained^32,44^.

Our results show that at a volumetric flux of 56.5 $\hbox {L}/\hbox {m}^{2}\, \hbox {h}$, the highest mass of AR27 was removed, and the specific AR27 biosorption capacity and volumetric AR27 biosorption capacity values were maximal; therefore, subsequent studies were carried out using an influent solution with a volumetric flux of 56.5 $\hbox {L}/\hbox {m}^{2}\, \hbox {h}$.

### Effect of inlet AR27 concentration on dye biosorption

Figure 3C shows the AR27 biosorption breakthrough curves obtained with different inlet AR27 concentrations, ranging from 30 to 400 mg/L (30, 50, 75, 100, 200, and 400 mg/L), when the inlet pH, volumetric flux of the influent solution, and LEC bed height remained constant at 2.0, 56.5 $\hbox {L}/\hbox {m}^{2}\, \hbox {h}$, and 2 cm, respectively.

At influent AR27 concentrations of 30, 50, 75, 100, and 200 mg/L, the column effluents did not contain a detectable amount of AR27 dye during the first 60, 45, 30, 21 and 6 h of column operation, respectively, and all the dye initially present in the inlet solutions was biosorbed. In contrast, AR27 dye was found in the outflow effluent from the start of operation of the packed-bed column, when an inlet solution with an AR27 concentration of 400 mg/L was fed to the column. These results clearly show that the service time of the column increased with a decrease in the influent AR27 concentration.

Similarly, the slope of the breakthrough curves increased as the concentration of influent AR27 increased, which resulted in a decrease in column saturation time with an increase in the influent AR27 concentration.

It is evident that both the service time and saturation time of the packed-bed column were lower at higher influent AR27 concentrations. This can be explained by the fact that the higher the influent AR27 concentration, the greater the AR27 concentration gradient, and consequently, the greater the thermodynamic driving force for dye biosorption. Thus, the AR27 molecules are biosorbed more quickly onto the biosorbent when the dye concentration is higher; consequently, the service and saturation time periods are minimal^32^.

Qualitatively similar behavior was reported in previous packed-bed column studies on the biosorption of methylene blue by *Enteromorpha prolifera*^49^ , *Eucalyptus sheathiana *bark^50^, and pyrolytic tire char^30^, crystal violet by *Citrullus lanatus* rind and *Cyperus rotundus*^51^, direct blue 71 by cross-linked chitosan-glutaraldehyde beads^29^, and reactive red 198 by activated carbon developed from walnut shells^52^.

The maximum specific and volumetric biosorption capacities of AR27 were observed at inlet AR27 concentrations ranging from 50 to 400 mg/L ($\hbox {p} > 0.05$), and were approximately 66 mg/g and 12,390 mg/L, respectively (Table 4). The maximal specific biosorption capacity for AR27 in the continuous LEC packed-bed system is similar to that achieved in a batch system (69.1 mg/g)^25^, and is considerably higher than those reported in a few studies on batch AR27 adsorption, including those using peanut hull^53^, de-oiled soya, bottom ash^54^, raw and $\hbox {H}_{3}\hbox {PO}_{4}$-pretreated pineapple peels and coconut shells^55^, alumina reinforced polystyrene^24^, dull pink clay^8^, polyethyleneimine grafted chitosan beads^14^, activated carbon web produced from acrylic fibrous waste^26^, $\hbox {Fe}_{3}\hbox {O}_{4}/\hbox {MgO}$ nanoparticles^28^, and *Arachis hypogaea* shells^27^ as adsorbents.

**Table 4:** Kinetic parameter values from various breakthrough curve models for AR27 biosorption onto LEC using influent solutions with different AR27 concentrations.

	AR27 concentration (mg/L)					
	30	50	75	100	200	400
**Experimental**						
$\hbox {m}_{\mathrm{r}}$ (mg)	27.78 ± 1.39	33.63 ± 1.68	34.75 ± 1.7	32.13 ± 1.61	30.25 ± 1.51	33.1 ± 1.65
q (mg/g)	55.55 ± 2.78	67.25 ± 3.36	69.5. ± 3.47	64.25 ± 3.21	60.5 ± 3.05	66.2 ± 3.33
$\hbox {N}_{\mathrm{v}}$ (mg/L)	10,484 ± 524.2	12,611 ± 630.5	13,052 ± 652.6	12,009 ± 600.4	11,378 ± 568.9	12,892 ± 644.6
$\tau$ (h)	79 ± 3.95	63 ± 3.15	44 ± 2.2	29.5 ± 1.47	13.5 ±0.67	7.7 ± 0.38
**Thomas**						
q (mg/g)	55.88 ± 0.09	66.92 ± 0.14	69.25 ± 0.14	64.75 ± 0.13	61.86 ± 0.30	67.86 ± 0.95
$\hbox {k}_{\mathrm{Th}}$ (L/mg h)	0.004 ± 0.001	0.004 ± 0.001	0.003 ± 0.001	0.003 ± 0.001	0.002 ± 0.001	0.001 ± 0.001
$\hbox {R}^{2}$	0.995	0.996	0.997	0.998	0.994	0.988
RMSE	0.281	0.272	0.277	0.184	0.259	0.155
AIC	− 2374	− 2940	− 3778	− 3985	− 3810	− 822.8
**Bed depth service time (BDST)**						
$\hbox {N}_{\mathrm{vBDST}}$ (mg/L)	10,520 ± 16.87	12,597 ± 19.37	13,037 ± 26.3	12,209 ± 24.84	11,664 ± 55.92	12,795 ± 178.9
$\hbox {k}_{\mathrm{BDST}}$ (L/ mg h)	0.004 ± 0.001	0.004 ± 0.001	0.003 ± 0.001	0.003 ± 0.001	0.002 ± 0.001	0.001 ± 0.001
$\hbox {R}^{2}$	0.995	0.995	0.997	0.998	0.994	0.988
RMSE	0.281	0.187	0.277	0.183	0.259	0.155
AIC	− 2374	− 2791	− 3778	− 3985	− 3810	− 822.8
**Yoon–Nelson**						
$\tau _{\mathrm{YN}}$ (h)	79.73 ± 0.20	62.55 ± 0.14	43.78 ± 0.12	29.83 ± 0.09	13.95 ± 0.01	7.977 ± 0.16
$\hbox {k}_{\mathrm{YN}}$ (1/h)	0.129 ± 0.003	0.196 ± 0.005	0.215 ± 0.004	0.284 ± 0.006	0.317 ± 0.089	0.3085 ± 0.014
$\hbox {R}^{2}$	0.995	0.996	0.997	0.998	0.994	0.988
RMSE	0.281	0.272	0.277	0.183	0.259	0.155
AIC	− 2374	− 2940	− 3778	− 3985	− 3810	− 822.8
**Modified dose-response**						
$\tau _{\mathrm{DR}}$ (h)	79.19 ± 0.18	62.31 ± 0.15	43.39 ± 0.19	29.51 ± 0.08	13.46 ± 0.53	7.56 ± 0.32
$\alpha$	10.28 ± 0.205	12.1 ± 0.32	9.41 ± 0.19	8.46 ± 0.18	4.36 ± 0.06	2.29 ± 0.30
$\hbox {R}^{2}$	0.996	0.996	0.998	0.998	0.998	0.942
RMSE	0.218	0.240	0.214	0.168	0.073	0.750
AIC	− 2460	− 2231	− 3907	− 4029	− 4449	− 627.2
**Logistic equation**						
a	10.22 ± 0.24	12.08 ± 0.28	9.40 ± 0.22	8.46 ± 0.19	4.42 ± 0.13	2.46 ± 0.12
b	0.128 ± 0.003	0.193 ± 0.005	0.215 ± 0.005	0.283 ± 0.006	0.317 ± 0.009	0.308 ± 0.014
$\hbox {R}^{2}$	0.9949	0.9968	0.9972	0.9977	0.9937	0.9881
RMSE	0.2815	0.1874	0.2766	0.1834	0.2595	0.1548
AIC	− 2374	− 2308	− 3778	− 3985	− 3810	− 822.8

### Influence of LEC bed height on AR27 biosorption

In order to evaluate the performance of the LEC biomass for AR27 biosorption at different bed heights in the packed-bed bioreactor, 0.5, 1, 1.5, and 2 g of LEC biomass were placed in the column to obtain bed heights of 2, 4, 6, and 8 cm, respectively. Experiments were carried out using influent solution pH, volumetric flux, and AR27 concentration of 2.0, 56.5 $\hbox {L}/\hbox {m}^{2}\, \hbox {h}$, and 200 mg/L, respectively.

Figure 3D displays the breakthrough profiles of AR27 biosorption at various bed heights. It is apparent that the slope and shape of the breakthrough curves differ with a variation in the LEC bed height.

The effluents were found to be free of AR27 dye during the first 6, 20, 37, and 43 h of packed-bed column operation, when the bed heights were 2, 4, 6, and 8 cm, respectively. At operation times longer than those mentioned above and at all the LEC bed heights tested, the effluent AR27 concentration increased as the operation time of the column progressed, which suggests a greater depletion of the active sites for dye biosorption on the LEC surface with an increase in the operating time.

As the height of the biosorbent bed increased, the slope of the breakthrough curves decreased, which resulted in a decrease in effluent dye concentrations, and the exhaustion of the LEC packed bed was slower. Consequently, the saturation time of the column increased. These results are consistent with those presented by other researchers who studied the packed-bed column biosorption of methyl violet 2B onto *Carya illinoensis* pericarp^56^, congo red onto Bengal gram seed husk^57^, remazol reactive dyes onto *R. arrhizus* NCIM 997^46^, yellow dye tartrazine onto *M. oleifera*^45^, and direct blue 71 onto cross-linked chitosan-glutaraldehyde beads^29^.

In addition, Table 5 shows that when the bed height increased, the mass of the AR27 dye removed ($\hbox {m}_{\mathrm{r}}$) by LEC increased; this could be attributed to the fact that the greater the bed height, the larger the mass of biosorbent in the column that augments the surface area and the number of active binding sites available for AR27 biosorption. Furthermore, the hydraulic retention time for the AR27 solution in the column increased with an increase in the bed height, allowing AR27 molecules to be in contact with the LEC biomass for a longer period and diffuse deeper into the biosorbent, which resulted in the removal of a larger mass of dye by the LEC packed bed. However, no statistically significant differences were observed in the specific and volumetric AR27 biosorption capacities observed with different bed heights, which indicates that the amount of AR27 removed is directly proportional to the amount of LEC biomass packed in the bed.

**Table 5:** Kinetic parameter values from various breakthrough curve models for AR27 biosorption onto LEC at different LEC bed heights.

	LEC bed height (cm)			
	2	4	6	8
**Experimental**				
$\hbox {m}_{\mathrm{r}}$ (mg)	34.75 ± 1.74	67.25 ± 3.36	96 ± 4.8	131.5 ± 6.57
q (mg/g)	69.5 ± 3.47	67.25 ± 3.36	64.0 ± 3.2	65.75 ± 6.57
$\hbox {N}_{\mathrm{v}}$ (mg/L)	13,052 ± 652.6	12,605 ± 630.25	12,083 ± 604.15	12,428 ± 621.4
$\tau$ (h)	14 ± 2.2	31.5 ± 1.57	44.0 ± 2.2	61 ± 3.05
**Thomas**				
q (mg/g)	69.25 ± 0.14	67.33 ± 0.22	64.26 ± 0.15	66.16 ± 0.11
$\hbox {k}_{\mathrm{Th}}$ (L/mg h)	0.003 ± 0.001	0.002 ± 0.001	0.002 ± 0.001	0.001 ± 0.001
$\hbox {R}^{2}$	0.997	0.993	0.994	0.995
RMSE	0.277	0.238	0.471	0.308
AIC	− 3778	− 1622	− 2323	− 2570
**Bed depth service time (BDST)**				
$\hbox {N}_{\mathrm{vBDST}}$ (mg/ L)	13,037 ± 26.3	12,674 ± 41.1	12,097 ± 27.92	12,459 ± 21.08
$\hbox {k}_{\mathrm{BDST}}$ (L/mg h)	0.003 ± 0.001	0.001 ± 0.001	0.002 ± 0.001	0.001 ± 0.001
$\hbox {R}^{2}$	0.997	0.993	0.994	0.995
RMSE	0.277	0.238	0.471	0.308
AIC	− 3778	− 1622	− 2323	− 2570
**Yoon–Nelson**				
$\tau _{\mathrm{YN}}$ (h)	13.78 ± 0.12	31.55 ± 0.14	44.26 ± 0.15	61.58 ± 0.15
$\hbox {k}_{\mathrm{YN}}$ (1/h)	0.215 ± 0.004	0.289 ± 0.01	0.367 ± 0.018	0.226 ± 0.007
$\hbox {R}^{2}$	0.997	0.993	0.994	0.995
RMSE	0.277	0.238	0.471	0.308
AIC	− 3778	− 1622	− 2323	− 2570
**Modified dose-response**				
$\tau _{\mathrm{DR}}$ (h)	13.39 ± 0.19	31.25 ± 0.14	44.18 ± 0.13	61.31 ± 0.15
$\alpha$	9.41 ± 0.19	9.134 ± 0.33	15.87 ± 0.66	14.14 ± 0.42
$\hbox {R}^{2}$	0.998	0.993	0.995	0.995
RMSE	0.214	0.228	0.369	0.303
AIC	− 3907	− 1632	− 2408	− 2575
**Logistic equation**				
a	9.40 ± 0.22	9.12 ± 0.34	16.26 ± 0.78	14.1 ± 0.43
b	0.214 ± 0.005	0.289 ± 0.005	0.367 ± 0.018	0.229 ± 0.007
$\hbox {R}^{2}$	0.997	0.993	0.994	0.995
RMSE	0.277	0.238	0.471	0.308
AIC	− 3778	− 1622	− 2323	− 2570

### Modeling of the AR27 biosorption breakthrough curves

The successful optimization of the design and operating conditions of a packed-bed column biosorption process requires mathematical models that can predict the breakthrough curves for the effluent.

The Thomas, Yoon–Nelson and Bohart–Adams models (or its variant, the bed depth service time (BDST) model) are the most commonly used mathematical models for the analysis of the dynamic behavior of the packed-bed column in the adsorbate-adsorbent system. It has been argued that all these models can be represented using the logistic model of population growth with two general parameters^42^.

In this work, the BDST, Thomas, modified dose-response, Yoon–Nelson, and logistic models were adjusted to the experimental breakthrough data to describe the dynamic performance of the packed-bed column reactor at various pHs, volumetric fluxes, AR27 concentrations and LEC bed heights.

Tables 2, 3, 4 and 5 display the parameter values of kinetic models of the breakthrough curves for AR27 biosorption, as well as the corresponding values of $\hbox {R}^{2}$, RMSE, AIC, and 95% confidence interval.

The rate constant $\hbox {k}_{\mathrm{Th}}$ of the Thomas model, which characterizes the rate of adsorbate transfer from the liquid to the solid phase, showed no significant difference at influent solution pH values ranging between 1.5 and 4.0, but increased as the inlet solution pH increased from 4.0 to 6.0. All influent volumetric fluxes assayed showed $\hbox {k}_{\mathrm{Th}}$ values not to be significantly different, but decreased when both inlet AR27 concentration and LEC bed height were increased, which indicates that external diffusion is not the controlling factor in the AR27 biosorption process^50^.

The q values predicted by the Thomas model were higher at solution pHs of 1.5 and 2.0 and at volumetric fluxes of 37.67 and 56.5 $\hbox {L}/\hbox {m}^{2}\, \hbox {h}$, but decreased when solution pH was increased from 2.0 to 6.0 and volumetric flux from 56.5 to 113 $\hbox {L}/\hbox {m}^{2}\, \hbox {h}$.

Likewise, all LEC bed heights assayed showed q values not to manifest significant differences and nor did influent AR27 concentrations ranging between 50 and 400 mg/L. The Thomas model is apt for describing biosorption processes, where external and internal resistance to the mass transfer of solute from the liquid to the solid phase is extremely low, and likewise biosorption is not limited by the chemical reaction, but is controlled by mass transfer at the interface^30^.

The rate constant ($\hbox {k}_{\mathrm{BDST}}$) of the BDST model characterizes the rate of solute transfer from the liquid phase to the solid phase^50^. The $\hbox {k}_{\mathrm{BDST}}$ values were not significantly different at inlet solution pHs ranging between 1.5 and 4.0, but increased when solution pH was increased from 4.0 to 6.0. For the AR27 volumetric fluxes assayed, the $\hbox {k}_{\mathrm{BDST}}$ values showed no significant difference, but this decreased when both the influent dye concentration and the biosorbent bed height were increased. These results indicated that overall system kinetics was not dominated by external mass transfer^30,50^. The volumetric biosorption capacity $\hbox {N}_{\mathrm{vBDST}}$ predicted by the BDST model was greater at influent solution pHs of 1.5 and 2, and volumetric fluxes of 37.67 and 56.5 $\hbox {L}/\hbox {m}^{2}\, \hbox {h}$, but decreased when solution pH was increased from 2.0 to 6.0 and volumetric flux from 56.5 to 113 $\hbox {L}/\hbox {m}^{2}\, \hbox {h}$. Neither did $\hbox {N}_{\mathrm{vBDST}}$ values change significantly with the increase of the influent AR27 concentration and LEC bed height. The BDST model assumes that forces like intraparticle diffusion and external mass transfer resistance are negligible and that the adsorbate is directly adsorbed onto the biosorbent surface^51^.

The 50% breakthrough time $\tau _{\mathrm{YN}}$, forecast using the Yoon–Nelson model, was higher at influent pH solution values of 1.5 and 2 but decreased as solution pH increased from 2 to 6.0. $\tau _{\mathrm{YN}}$ values decreased when both influent volumetric flux and inlet AR27 concentration increased because saturation of the column occurred more rapidly but increased with LEC bed height increase. Furthermore, the $\hbox {k}_{\mathrm{YN}}$ rate constant from the Yoon–Nelson model did not show significant difference at influent solution pH values ranging between 1.5 and 4 but increased when solution pHs were increased from 4.0 to 6.0, as it did when volumetric flux, influent concentration, and bed height were increased. These results concur with those reported by Patel^58^, Han et al.^59^, Afroze et al.^50^, and Chen et al.^60^. The Yoon–Nelson model assumes that the decline in probability for each adsorbate to be biosorbed is proportional to the probability of its biosorption and breakthrough on the biosorbent^37^.

The kinetic constants of the modified dose-response model ($\tau _{\mathrm{DR}}$ and $\alpha$) were highest at influent solution pH values of 1.5 and 2.0 but decreased as solution pH increased from 2.0 to 6.0. Similarly, $\tau _{\mathrm{DR}}$ and $\alpha$ decreased, when both volumetric flux and influent concentration were increased, but increased when bed height was increased. The dose-response model has frequently been applied to represent pharmacokinetic processes and is presently used to describe biosorption of heavy metals and dyes in packed-bed columns^32,37^.

The kinetic constant a of the logistic model was higher at influent solution pHs of 1.5 and 2.0 but decreased as solution pH increased from 2.0 to 6.0. The a constant also decreased, when both the volumetric flux and the influent concentration were increased, but increased as the bed height increased. No significant difference was observed in the values for the b constant of the logistic model within a pH range for the influent solution of 1.5 and 4.0, but increased as solution pH increased from 4.0 to 6.0, as well as when volumetric flux, influent concentration, and bed height were increased.

The above results clearly indicate that lower influent solution pHs, lower-middle influent volumetric fluxes, middle-higher influent AR27 concentrations and higher bed heights would increase the mass of AR27 dye biosorbed on the LEC packed-bed.

It was also found that the BDST, Thomas, and Yoon–Nelson models had the same fitting effect as the logistic model, and were therefore mathematically equivalent. Similarly, it was observed that these four models and the modified dose-response model exhibited values of $R^{2}$, RMSE, and AIC that were very close to each other. Furthermore, the BDST, Thomas, Yoon–Nelson, and dose-response models were able to adequately predict the experimental values of q, $\hbox {N}_{\mathrm{v}}$, $\tau$ and $\tau$, respectively (Tables 2, 3, 4, 5). It was confirmed that upon using the values of the constants a and b in the logistic model along with the values of other operational and system variables, the kinetic parameters of the BDST, Thomas, and Yoon–Nelson models could be calculated from the mathematical relationships provided in Table 1^42^.

The above results indicate that the Thomas, BDST, Yoon–Nelson, logistic, and modified dose-response models were able to represent the behavior of the breakthrough curves for AR27 biosorption by the LEC packed bed.

Reck et al. ^45^ also found that the Thomas, Adams-Bohart, and dose-response kinetic models were able to reproduce the experimental data of tartrazine biosorption in a packed-bed column. Experimental results and mathematical modeling suggest that internal and external diffusion are not the controlling steps in the AR27 biosorption by LEC but is controlled by the mass transfer at the interface.

## Conclusions

In the present study, the LEC particles were characterized in terms of average specific surface area, particle size, pore size, zeta potential, and zero-charge point. Likewise, the porosity and bulk density of LEC packed-bed were determined. The biosorptive effectiveness of sustainable and cost-effective LEC biomass in the biosorption of AR27 from aqueous solutions was assessed using a continuous up-flow packed-bed column reactor. The AR27 biosorption in the continuous biosorption system depends on the pH, volumetric flux, and AR27 concentration of the influent AR27 solution, as well as on the LEC bed height. The packed-bed biosorption system was found to perform better when operated with an influent solution pH of 2.0, a volumetric flux of inlet solution of 56.5 $\hbox {L}/\hbox {m}^{2}\, \hbox {h}$, and with influent AR27 concentrations and LEC bed heights ranging between 50 and 400 mg/L and 2 and 8 cm, respectively. Furthermore, it was observed that the lower the volumetric flux and the AR27 concentration of the influent solution, the greater the service and saturation time of the packed-bed column. Similarly, the higher the LEC bed height, the greater the service and saturation time of the packed-bed column. The breakthrough curves for AR27 biosorption onto LEC were mathematically modeled and the kinetic biosorption parameters were determined. The Thomas, BDST, Yoon–Nelson, modified dose-response and logistic models accurately described the entire breakthrough curves, at all the operating conditions assayed. The results obtained demonstrate that LEC is a highly attractive biomaterial for the effective, continuous bioremediation of AR27-contaminated aqueous solutions in a packed-bed column system.
